# GP referrals for suspected cancer in Ireland: protocol for a cross-sectional study (GRACCHUS)

**DOI:** 10.12688/hrbopenres.14109.1

**Published:** 2025-04-09

**Authors:** Katie Killeen, Sean O'Regan, Conor F. Murphy, Benjamin Jacob, Yiren Yin, Heather Burns, Kathleen Bennett, Patrick Redmond

**Affiliations:** 1 Department of General Practice, Royal College of Surgeons in Ireland (RCSI), Dublin, Ireland; 2 National Cancer Control Programme, Health Service Executive, Dublin, Ireland; 3 Data Science Centre, School of Population Health, Royal College of Surgeons in Ireland (RCSI), Dublin, Ireland

**Keywords:** Primary Health Care; Early Detection of Cancer; Referral and Consultation; Diagnostic Services; Cross-Sectional Studies; Ireland.

## Abstract

**Background:**

Cancer is a leading cause of mortality in Ireland, accounting for approximately 30% of deaths annually. Early diagnosis improves survival, reduces treatment burden, and enhances patient outcomes. Rapid Access Clinics (RACs) were introduced to facilitate expedited diagnosis of suspected lung, prostate, and breast cancers, as well as malignant melanoma. However, the extent to which Irish general practitioners (GPs) utilise RAC pathways, and the subsequent diagnostic outcomes, remain poorly understood.

**Methods:**

This retrospective repeated cross-sectional study will analyse electronic health records from Irish general practices (2013–2024). Phase 1 will assess trends in RAC referrals, including volume, cancer type, and inter-practice variation, alongside demographic, geographic, and clinical factors influencing referral rates. Phase 2 will evaluate cancer conversion rates, time to diagnosis, stage at diagnosis, treatment received, and cancer-specific mortality. Data collection will use a validated extraction tool, and analysis will follow STROBE guidelines for observational studies.

**Expected Outcomes:**

This study will quantify RAC referral patterns and identify factors influencing variability in GP referral behaviour. It will also assess diagnostic yield and cancer outcomes associated with RAC referrals. Findings will inform quality improvement initiatives and policy development to optimise cancer diagnostic pathways in Ireland.

## Introduction

### Cancer burden and the importance of early diagnosis

Cancer remains a leading cause of mortality worldwide, accounting for one in six deaths ^1^. In Ireland, approximately 43,000 people are diagnosed with cancer each year, with over 9,000 cancer-related deaths, representing nearly one-third of all national mortality ^2^. Lung cancer is the most common cause of cancer death in Ireland, responsible for 20% of cancer-related mortality, followed by breast cancer in women, prostate cancer in men, and colorectal cancer in both sexes ^2^.

Cancer survival is influenced by multiple factors, including tumour biology, patient comorbidities, and access to effective treatments. However, for most tumour types, stage at diagnosis is the strongest predictor of survival ^3^. In Ireland, 33% of lung cancers are diagnosed at stage I or II, compared to 45% of colorectal cancers and 79% of breast cancers. Five-year net survival for lung cancer is just 24%, compared to 66% for colorectal cancer and 88% for breast cancer ^2, 4^. Given that early-stage cancers are more amenable to curative treatment, strategies that facilitate timely diagnosis are critical for improving patient outcomes and reducing healthcare burden ^3, 5, 6^.

### Rapid Access Clinics in Ireland: structure, utilisation, and limitations

The National Cancer Control Programme (NCCP) was established in 2007 as a directorate of the Health Service Executive (HSE) to coordinate cancer services, standardise care, and improve survival rates. A key initiative has been the development of Rapid Access Clinics (RACs), specialist-led services designed to expedite diagnosis and specialist assessment for suspected breast, lung, prostate cancer, and melanoma—the four most commonly diagnosed cancers in Ireland ^7^.

RACs operate within designated cancer centres, with defined targets for appointment scheduling, aiming to ensure timely evaluation following GP referral ^4^. International evidence suggests that structured pathways such as RACs improve access to diagnostic services, reduce diagnostic delays, and increase the proportion of cancers diagnosed at an early stage ^5, 8^. Despite this, Irish data suggest that diagnostic pathways may not be fully optimised, as a significant proportion of cancers continue to be diagnosed at an advanced stage ^4^.

### Variation in GP referral behaviour and patient outcomes

General practitioners (GPs) are central to early cancer detection, with the majority of symptomatic cancer patients initially presenting in primary care ^8^. However, referral pathways are complex, and the decision to refer is influenced by clinical guidelines, GP experience, patient characteristics, and healthcare system constraints ^9, 10^. While RACs provide an expedited pathway for specialist assessment, referral decisions are complex, and substantial variation in GP referral behaviour has been documented in international studies ^11^.

In Denmark, where structured urgent referral pathways have been in place since 2008, approximately 27% of lung cancer diagnoses occur via fast-track pathways ^8^. However, GP referral rates for suspected cancer vary significantly between practices, even after adjusting for case-mix, practice size, and patient demographics ^11– 13^. Notably, higher referral rates are associated with lower emergency presentation rates and improved cancer survival ^14^. These findings suggest that greater utilisation of structured referral pathways may contribute to earlier cancer detection and better patient outcomes.

In contrast, there is limited evidence on the extent of referral variation within Ireland and its impact on cancer diagnosis and outcomes. The "Cancer Care in Ireland 2020" report recorded 44,245 new attendances at RACs for breast, prostate, and lung cancers in a single year ^15^. However, the conversion rate (proportion of referrals resulting in a cancer diagnosis) remains unclear, making it difficult to assess the efficiency of the RAC system. Additionally, how referral rates vary by patient demographics, GP practice characteristics, and geographic location has not been explored, limiting opportunities for targeted improvements in diagnostic pathways.

Given the established link between referral patterns and cancer outcomes ^5, 14^, a detailed examination of RAC referral trends is necessary to identify potential barriers to timely cancer diagnosis. These may include regional disparities in access to diagnostic services, differences in GP decision-making and adherence to referral guidelines, and variations in patient presentation, such as symptom severity and comorbidities. Furthermore, analysing the relationship between referral rates and conversion rates will provide a clearer picture of how effectively RAC pathways function in practice. Identifying patterns of under-referral or over-referral will be essential for improving diagnostic efficiency, ensuring that referral decisions align with clinical need. This evidence will inform GP education and the refinement of clinical guidelines to promote appropriate referrals, support policy interventions aimed at addressing gaps in cancer diagnostic services, and enhance quality improvement initiatives to optimise the equitable and efficient use of RACs.

This study is the first in Ireland to propose a comprehensive, retrospective cross-sectional analysis of RAC referrals, using electronic health records (EHRs) from general practice. By quantifying referral trends, identifying predictive factors, and assessing diagnostic outcomes, this research will generate critical evidence to strengthen RAC pathways and improve early cancer detection.

### Aims and objectives

This study aims to examine general practice referrals to RACs for suspected cancer in Ireland, evaluating referral patterns, variation across practices, and the impact on cancer diagnosis and outcomes. Specific objectives include:

•   To characterise RAC referral patterns by cancer type, volume, and inter-practice variability over time.

•   To examine demographic, geographic, and clinical factors influencing referral rates.

•   To determine the diagnostic conversion rate, time from referral to diagnosis, and stage at diagnosis for patients referred to RACs.

•   To assess cancer treatment and survival outcomes following RAC referral.

## Methods

### Study design

This is a retrospective study that will analyse electronic health record (EHR) data from Centric Health’s network of general practices in Ireland. Specifically, we will examine electronic rapid access cancer referrals for breast, prostate, lung cancer, and melanoma, by GPs in Ireland from 2013–2024. The data collection and analysis for the study will be conducted throughout 2025. The study will be conducted in two phases.

The first phase has three steps: 1) a descriptive analysis of utilisation of RAC pathways over time and how this varies by GP practice; 2) a series of descriptive analyses, which, for each referral type, examines how the prevalence or degree of a referral characteristic varies over time, and 3) a series of negative binomial regression analyses which seek to explain the variation in the number of referrals (of a particular type) from a GP practice varies as a function of GP practice characteristics, patients characteristics and time.

The second phase involves: 1) a descriptive analysis of outcomes of RAC referrals over time, and 2) an exploration and analysis of characteristics predictive of cancer diagnosis and outcomes over time. We will adhere to the SPIROS (Standardized Protocol Items Recommendations for Observational Studies) and STROBE (Strengthening the Reporting of Observational Studies in Epidemiology) Checklist for reporting protocols and cross-sectional studies, respectively ^16, 17^.

### Study setting & participants

The setting includes GP practices from the Centric Health practice network. Eligible participating practices are GP practices utilising the Socrates patient management system (Socrates Healthcare Ltd. Sligo, Ireland; https://www.socrates.ie/). Participation will be discussed with practices from the targeted networks after outlining the study's scope and significance. These inclusion criteria are designed to ensure a reliable dataset and consistency in data collection methods.

### Sample size

As this is an observational study of a general practice population that aims to characterise current practice, and generate hypotheses for further investigation, there is no intent to conduct inferential analyses beyond the studied population. As such, a sample size calculation is not necessary.

### Variables

The primary outcome variable for phase 1 is:

1) The RAC pathway referral volume & rate, standardised for age and sex distribution, by cancer type, and variation over time and between practices.
2) Secondary outcomes will include exploration of practice and patient characteristics that predict referral rates over time.

The primary outcome variable for phase 2 is:

1) The conversion rate, defined as the proportion of referrals resulting in a cancer diagnosis, by cancer type, and variation over time and between practices.
2) Secondary outcomes will include cancer stage at diagnosis, time from referral to diagnosis, treatment received, and mortality for each cancer diagnosis, as well as characterisation of practice and patient factors that predict conversion rate for each cancer type.

### Data collection and management

***Data extraction tool***

A data extraction tool, based on the Visual Basic for Applications (VBA) language was designed for phase 1 of this study (Microsoft. (2025). Microsoft Office [Visual Basic for Applications]. Available from https://www.microsoft.com/en-us/microsoft-365). It was designed to extract data in Extensible Markup Language, which is the output produced for referrals in some EHRs in Ireland. It then converts this data into a format suitable for tabulation and analysis in Microsoft Excel. It will collect variables as outlined in Table 1. The tool will be refined through a trial run of pilot practices with iterative debugging. Validity of the data extracted will be determined by comparing the proportion of records extracted relative to the known number of records. A successful extraction rate, indicating adequate completeness of data, namely complete extraction of information from a referral file, will be defined as exceeding a threshold of 95%.

**Table 1.** Overview of planned data collection & data source.

Data Item	Description	Format/Type	Source
**Patient Characteristics**			
**Patient Age/DOB**	Age & DOB of the patient	Numerical/Date	GP records
**Referral Type**	Type of cancer referral	Categorical	GP records
**Sex**	Gender of the patient	Categorical	GP records
**Clinical Info**	Relevant clinical information	Text	GP records
**Referral Date**	Date when the referral was made	Date	GP records
**GMS Status**	Private or General Medical Services card holder (GMS)	Categorical	GP records
**Practice Characteristics**			
**Practice Location**	Geographic location of the practice	Categorical	Eircode.ie
**Practice Size**	Number of GP sessions per week worked in the practice	Numerical	Practice records
**Practice Size**	Number of patients registered to the practice	Numerical	Practice records
**GMS List size**	Proportion of GMS patients relative to total active patients	Numerical	Practice records
**Deprivation index**	Quantitative measure of socioeconomic status of population	Numerical	Pobal HP deprivation index based on Eircode
**Practice population density**	Urban vs rural	Categorical	Pobal HP / Practice Eircode
**Patient Outcomes**			
**Cancer diagnosis**	Was there an ultimate cancer diagnosis?	Categorical	GP records
**Cancer investigations and diagnosis timeline**	Date of RACC investigations and cancer diagnosis.	Freetext	GP records
**Cancer stage**	Stage of cancer at diagnosis	Categorical	GP records
**Cancer treatment**	Type of treatment received. Date of treatment commencement.	Categorical	GP records
**Cancer mortality**	Did the patient die of cancer? Date of death.	Categorical	GP records

***Electronic Health Record chart review***

Patient EHRs will be manually searched by the study team and medical students under the supervision of Centric Health in each practice to extract the data for phase 2, specifically extraction the information listed in Table 1. The list of patient charts to review will be produced from the phase 1 data extraction process. Researchers will login to the EHR using a specific temporary login provided by the Practice Manager. This will have an associated audit log providing oversight of the data viewed during the chart review process. A Standard Operating Procedure (SOP) for the data collection process will be developed and used to train the relevant personnel, ensuring consistency and quality of data throughout phase 2. Sample data collection spreadsheets will be freely available via Open Science Framework.

***Data governance***

Data will be pseudonymised following extraction from the clinical records in Phase 1, ensuring the removal of identifiable personal information. The pseudonymised data will remain on the same secure server in a password-protected document with access strictly limited to authorised members of the research team. The unique identifier linking this data to identifiable patient information will be stored separately in a secure, password-protected file.

***Bias***

Potential sources of bias include recruitment from a specific network of practices, and exclusion of non-Centric health practices. These will be addressed and quantified by ensuring a range of practices are included and by analysing the representativeness of participating practices. Efforts to mitigate bias will be documented and discussed in the study's findings.

### Ethics

Ethical approval for the study was granted by the Irish College of General Practitioners Research Ethics Committee (REC) on 12th December 2024 (reference no. 2662). According to the Amendments (January 2021) to the Health Research Regulations 2018, individual consent is not required for a low risk, retrospective chart review that has gained approval by the appropriate REC and is conducted by authorised parties ^16^. There is low risk of potential harm to participants of the study. There is no intent to involve the patients or the public in the design of this study protocol.

### Statistical approach

Data analysis for phase 1 of the study consists of three parts focusing on GP practice characteristics, referral volume trends and clinical information trends respectively.

1) Initially, the characteristics of GP practices will be summarised, specifically the number of patients, age-sex distribution of patients, proportion of General Medical Services (GMS) patients, and the deprivation index and rurality of the practice location.
2) Referral trends between practices will be assessed by characterising the referral volume distribution across practices, year of referral, and time of year. Additionally, these results will be stratified by cancer type and adjusted first for GP practice size, and for age and sex, to investigate further associations or trends.
3) Clinical information of referred patients from all practices will be summarised, stratified by cancer type and year of referral to map trends for the population over time. Any inter-practice variation will then be characterised.

Data analysis for phase 2 of the study will focus on conversion rates for referrals as well as cancer stage at diagnosis and time from referral to diagnosis. First, conversion rates for all referrals over the study period will be presented by cancer type. Then rates will additionally be stratified by year of referral. The timeline from referral to diagnosis including pertinent investigations will be described for each cancer type for each practice. Finally, conversion rates will be characterised by all of cancer type, year of referral, and GP practice. The (TNM and/or Gleason) stage of cancer at diagnosis will be summarised for each cancer type.

## Discussion

This study aims to explore patterns in GP referrals for suspected cancer, stratified by practice characteristics, patient demographics and clinical features. It then aims to determine the rate of cancer diagnosis and to characterise diagnostic timelines and cancer outcomes. We anticipate that our findings will provide a detailed understanding of referral behaviours across various GP practices in Ireland and how these relate to cancer diagnosis and outcomes. By identifying specific trends and deviations, we will contribute to a nuanced understanding of how different factors, including geography, patient age, gender, and clinical presentations, influence the referral process and cancer outcomes.

### Implications for practice and policy

The anticipated results of this study are expected to provide a foundation for future research and inform policy and practice surrounding cancer diagnosis – particularly in primary care - in Ireland. We expect that the insights gained will be crucial in guiding population health policy, particularly in improving early detection strategies for cancer. By understanding referral patterns and associated cancer outcomes, participating GP practices can benchmark their behaviours against wider trends, potentially leading to quality improvement initiatives or an informed audit cycle.

### Limitations

While aiming for comprehensive and representative findings, we acknowledge potential limitations in our study. These may include the representativeness of the sample and inherent biases in referral practices. Moreover, the reliance on retrospective data from practices might introduce variability in the data quality. We plan to address these limitations through rigorous study design and transparent reporting of our methods and findings. Finally, while rapid access pathways are viewed as the primary route for referring patients with these suspected cancers, there are other potential routes of referral (paper-based or private referral pathways) that may not be captured in this dataset. However, the focus of this analysis is on the use of rapid access pathways rather than other routes to diagnosis.

### Dissemination

We plan a comprehensive dissemination strategy, targeting both academic and healthcare professional audiences. Findings will be submitted to peer-reviewed journals, presented at relevant conferences, and shared with healthcare policy makers. Additionally, we aim to engage with the wider public through media releases and open-access publications to ensure the study's implications are communicated broadly.

### Artificial Intelligence

Artificial intelligence (AI) assisted technologies have not been used in the preparation of this protocol and there is no intent to use them in the execution of this study.

## Conclusion

This study protocol describes an ambitious proposal to explore referral patterns for suspected cancer in Ireland and associated cancer outcomes. By providing a detailed and representative analysis, it aims to contribute valuable insights that will enhance understanding and inform practices and policies for better health outcomes.

## Ethics and consent

Ethical approval for the study was granted by the Irish College of General Practitioners Research Ethics Committee (REC) on 12th December 2024 (reference no. 2662). According to the Amendments (January 2021) to the Health Research Regulations 2018, individual consent is not required for a low risk, retrospective chart review that has gained approval by the appropriate REC and is conducted by authorised parties ^16^. There is low risk of potential harm to participants of the study. There is no intent to involve the patients or the public in the design of this study protocol.

## Data Availability

No data are associated with this article. **OSF:** GP Referrals for Suspected Cancer in Ireland: Protocol for a Cross-Sectional Study (GRACCHUS) DOI: https://doi.org/10.17605/OSF.IO/3S4P9 ^18^ The project contains the following extended data: GRACCHUS_EHR_DataExtraction_Tool.xlsx Data are available under the terms of the Creative Commons Attribution 4.0 International license (CC-BY 4.0). **OSF:** SPIROS checklist for ‘GP Referrals for Suspected Cancer in Ireland: Protocol for a Cross-Sectional Study (GRACCHUS)’ DOI: https://doi.org/10.17605/OSF.IO/3S4P9 ^18^ GRACCHUS_Protocol_SPIROS_checklist.pdf Data are available under the terms of the Creative Commons Attribution 4.0 International license (CC-BY 4.0).

## Software availability

N/A

## References

[1] GBD 2019 Diseases and Injuries Collaborators: Global burden of 369 diseases and injuries in 204 countries and territories, 1990–2019: a systematic analysis for the Global Burden of Disease Study 2019. *Lancet.* 2020;396(10258):1204–1222. 10.1016/S0140-6736(20)30925-9 33069326 PMC7567026

[2] National Cancer Registry Ireland: Cancer in Ireland 1994–2020: annual statistical report of the National Cancer Registry.[cited 2024 Sep 26]. Reference Source

[3] McPhail S Johnson S Greenberg D : Stage at diagnosis and early mortality from cancer in England. *Br J Cancer.* 2015;112 Suppl 1(Suppl 1):S108–S115. 10.1038/bjc.2015.49 25734389 PMC4385983

[4] National Cancer Control Programme: Early diagnosis of symptomatic cancer plan 2022 – 2025.2022. Reference Source

[5] Round T Gildea C Ashworth M : Association between use of urgent suspected cancer referral and mortality and stage at diagnosis: a 5–year national cohort study. *Br J Gen Pract.* 2020;70(695):e389–e398. 10.3399/bjgp20X709433 32312762 PMC7176359

[6] Neal RD Allgar VL Ali N : Stage, survival and delays in lung, colorectal, prostate and ovarian cancer: comparison between diagnostic routes. *Br J Gen Pract.* 2007;57(536):212–9. 17359608 PMC2042569

[7] Department of Health, Ireland: National cancer strategy 2017–2026.2017. Reference Source

[8] Guldbrandt LM Fenger-Grøn M Rasmussen TR : The role of general practice in routes to diagnosis of lung cancer in Denmark: a population-based study of general practice involvement, diagnostic activity and diagnostic intervals. *BMC Health Serv Res.* 2015;15: 21. 10.1186/s12913-014-0656-4 25608462 PMC4307896

[9] Baughan P Keatings J O’Neill B : Urgent suspected cancer referrals from general practice: audit of compliance with guidelines and referral outcomes. *Br J Gen Pract.* 2011;61(592):e700–e706. 10.3399/bjgp11X606591 22054333 PMC3207087

[10] Green T Atkin K Macleod U : Cancer detection in primary care: insights from general practitioners. *Br J Cancer.* 2015;112 Suppl 1(Suppl 1):S41–9. 10.1038/bjc.2015.41 25734388 PMC4385975

[11] Zhou Y Mendonca SC Abel GA : Variation in ‘fast-track’ referrals for suspected cancer by patient characteristic and cancer diagnosis: evidence from 670 000 patients with cancers of 35 different sites. *Br J Cancer.* 2018;118(1):24–31. 10.1038/bjc.2017.381 29182609 PMC5765227

[12] Round T Ashworth M L’Esperance V : Cancer detection via primary care urgent referral and association with practice characteristics: a retrospective cross-sectional study in England from 2009/2010 to 2018/2019. *Br J Gen Pract.* 2021;71(712):e826–e835. 10.3399/BJGP.2020.1030 34544690 PMC8463132

[13] Meechan D Gildea C Hollingworth L : Variation in use of the 2–week referral pathway for suspected cancer: a cross-sectional analysis. *Br J Gen Pract.* 2012;62(602):e590–7. 10.3399/bjgp12X654551 22947579 PMC3426597

[14] Møller H Gildea C Meechan D : Use of the English urgent referral pathway for suspected cancer and mortality in patients with cancer: cohort study. *BMJ.* 2015;351: h5102. 10.1136/bmj.h5102 26462713 PMC4604216

[15] Cancer care in Ireland in 2020: the impact of the COVID-19 pandemic.Royal College of Physicians of Ireland. [cited 2024 Sep 26]. Reference Source

[16] Vandenbroucke JP von Elm E Altman DG : Strengthening the Reporting of Observational Studies in Epidemiology (STROBE): explanation and elaboration. *Epidemiology.* 2007;18(6):805–35. 10.1097/EDE.0b013e3181577511 18049195

[17] Mahajan R Burza S Bouter LM : Standardized Protocol Items Recommendations for Observational Studies (SPIROS) for observational study protocol reporting guidelines: protocol for a Delphi study. *JMIR Res Protoc.* 2020;9(10): e17864. 10.2196/17864 33084592 PMC7641775

[18] Murphy CF Killeen K : GP referrals for suspected cancer in Ireland: protocol for a cross-sectional study (GRACCHUS). *OSF.* 2025. https://osf.io/3s4p9 10.12688/hrbopenres.14109.2 PMC12398676 40894358

