# Supplementary material for: GP referrals for suspected cancer in Ireland: protocol for a cross-sectional study (GRACCHUS)
Source: HRB Open Res. 2025 Aug 1;8:52. Originally published 2025 Apr 9. [Version 2] doi: 10.12688/hrbopenres.14109.2 (PMC12398676; doi:10.12688/hrbopenres.14109.2)
Supplement: Peer review history

# Reviewer report: Reviewer response for version 2

**DOI:** 10.21956/hrbopenres.15647.r48712

**Published:** 2025-08-30

**Authors:** Hein Van Poppel

**Affiliations:** 1 KU Leuven, Leuven, Flanders, Belgium

no further comments, happy with revised draft

Is the study design appropriate for the research question?

Yes

Is the rationale for, and objectives of, the study clearly described?

Yes

Are sufficient details of the methods provided to allow replication by others?

Partly

Are the datasets clearly presented in a useable and accessible format?

Yes

Reviewer Expertise:

Reduces Treatment Burden: It would be beneficial to state explicitly that early diagnosis enables earlier, less complicated, and less toxic treatment.Cancer Burden: While figures for lung, colorectal, and breast cancer are provided, it would enhance completeness to include corresponding data for prostate cancer, given its relevance to the studyVariation in GP Referral Behaviour: For prostate cancer, it should be clarified that in its curable stages, the disease is typically asymptomatic. Voiding symptoms are more commonly associated with benign prostatic hyperplasia (BPH), which affects referral behaviour differently than cancers presenting with clear symptoms.Limitations: The manuscript correctly highlights sample representativeness as a key limitation. However, further detail is needed on how the exclusion of handwritten referral letters will be mitigated, as this could significantly influence the national and international applicability of the findings. This is potentially a serious flaw, which could limit the usefulness of the data obtained in Phase 1 nationally and internationally in efforts to improve GP referrals and awareness of early cancer detection.Thank you for the opportunity to review this important work. I look forward to seeing how the project progresses.

I confirm that I have read this submission and believe that I have an appropriate level of expertise to confirm that it is of an acceptable scientific standard.

# Reviewer report: Reviewer response for version 1

**DOI:** 10.21956/hrbopenres.15506.r46763

**Published:** 2025-05-10

**Authors:** Alexander Ross Kerr

**Affiliations:** 1 New York University, New York, New York, USA

This retrospective cross-sectional study to review EHR data for selected GP practices to evaluate the efficiency of rapid access clinics for the early detection of the 4 most prevalent cancers in Ireland is well designed and an important study. One small criticism would be to focus on cancers with the poorest outcomes rather than the most common.

Is the study design appropriate for the research question?

Yes

Is the rationale for, and objectives of, the study clearly described?

Yes

Are sufficient details of the methods provided to allow replication by others?

Yes

Are the datasets clearly presented in a useable and accessible format?

Yes

Reviewer Expertise:

Oral cancer

I confirm that I have read this submission and believe that I have an appropriate level of expertise to confirm that it is of an acceptable scientific standard.

# Reviewer report: Reviewer response for version 1

**DOI:** 10.21956/hrbopenres.15506.r46756

**Published:** 2025-04-29

**Authors:** Hein Van Poppel

**Affiliations:** 1 KU Leuven, Leuven, Flanders, Belgium

I have reviewed the manuscript examining referral patterns from primary care to rapid access clinics for early cancer diagnosis. I would like to commend the team for addressing an important and underexplored area in Irish healthcare. All this is worthy of analysis and hasn’t been performed in Ireland.

 However I would like to raise a few points that may help strengthen the work further:

- **Reduces Treatment Burden**: It would be beneficial to state explicitly that early diagnosis enables earlier, less complicated, and less toxic treatment.
- **Cancer Burden**: While figures for lung, colorectal, and breast cancer are provided, it would enhance completeness to include corresponding data for prostate cancer, given its relevance to the study.
- **Variation in GP Referral Behaviour**: For prostate cancer, it should be clarified that in its curable stages, the disease is typically asymptomatic. Voiding symptoms are more commonly associated with benign prostatic hyperplasia (BPH), which affects referral behaviour differently than cancers presenting with clear symptoms.

**Limitations**: The manuscript correctly highlights sample representativeness as a key limitation. However, further detail is needed on how the exclusion of handwritten referral letters will be mitigated, as this could significantly influence the national and international applicability of the findings. This is potentially a serious flaw, which could limit the usefulness of the data obtained in Phase 1 nationally and internationally in efforts to improve GP referrals and awareness of early cancer detection.

 Thank you for the opportunity to review this important work. I look forward to seeing how the project progresses.

Is the study design appropriate for the research question?

Yes

Is the rationale for, and objectives of, the study clearly described?

Yes

Are sufficient details of the methods provided to allow replication by others?

Partly

Are the datasets clearly presented in a useable and accessible format?

Yes

Reviewer Expertise:

Reduces Treatment Burden: It would be beneficial to state explicitly that early diagnosis enables earlier, less complicated, and less toxic treatment.Cancer Burden: While figures for lung, colorectal, and breast cancer are provided, it would enhance completeness to include corresponding data for prostate cancer, given its relevance to the studyVariation in GP Referral Behaviour: For prostate cancer, it should be clarified that in its curable stages, the disease is typically asymptomatic. Voiding symptoms are more commonly associated with benign prostatic hyperplasia (BPH), which affects referral behaviour differently than cancers presenting with clear symptoms.Limitations: The manuscript correctly highlights sample representativeness as a key limitation. However, further detail is needed on how the exclusion of handwritten referral letters will be mitigated, as this could significantly influence the national and international applicability of the findings. This is potentially a serious flaw, which could limit the usefulness of the data obtained in Phase 1 nationally and internationally in efforts to improve GP referrals and awareness of early cancer detection.Thank you for the opportunity to review this important work. I look forward to seeing how the project progresses.

I confirm that I have read this submission and believe that I have an appropriate level of expertise to confirm that it is of an acceptable scientific standard, however I have significant reservations, as outlined above.
